# Supplementary material for: Food Categories for Breakfast and Mental Health among Children in Japan: Results from the A-CHILD Study
Source: Nutrients. 2023 Feb 22;15(5):1091. doi: 10.3390/nu15051091 (PMC10005218; doi:10.3390/nu15051091)
Supplement: Supplementary file 1 [file nutrients-15-01091-s001.zip › nutrients-2240495-supplementary.pdf]

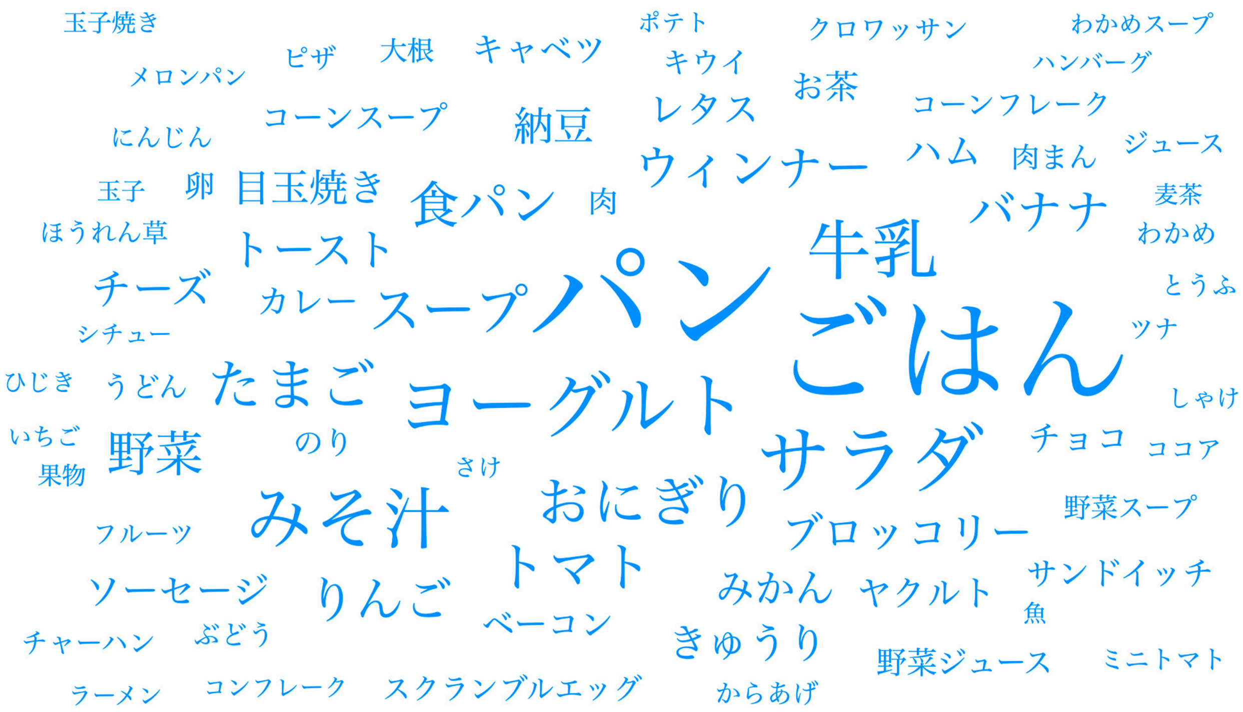

Figure S1. Word cloud for children's breakfast menus.

Table S1. Results of regression analyses on children's mental health according to types of grain dishes among Japanese school children (n = 281)

Food category	Behavior problems (SDQ score)	Prosocial behavior (SDQ score)
	coefficient (95%CI)	coefficient (95%CI)
Model 1		
Rice	-7.71 (-15.3 to -0.08)	-0.18 (-12.3 to 11.90)
Bread	-7.12 (-14.70 to 0.46)	1.99 (-10.0 to 14.00)
Model 2		
Rice	-10.9 (-19.9 to -1.8)	0.34 (-14.2 to 14.84)
Bread	-7.6 (-16.3 to 1.0)	0.48 (-13.3 to 14.31)
Vegetable dishes	0.91 (-4.52 to 6.34)	1.00 (-7.7 to 9.70)
Meat, fish, egg, and soy-bean dishes	0.01 (-6.25 to 6.27)	-5.69 (-15.7 to 4.35)
Milk and Milk products	-3.07 (-8.74 to 2.59)	-1.33 (-10.4 to 7.75)
Fruits	-7.07 (-13.8 to -0.37)	7.28 (-3.4 to 18.02)
Confectionaries	-2.00 (-11.4 to 7.45)	-4.95 (-20.1 to 10.19)

CI, confidence interval; SD, standard deviation; SDQ, Strength and Difficulties Questionnaire.

All variables ranged from 0 to 100.

Model 1: Inclusion of rice and bread dishes with adjustment for child's sex, physical activity, and bedtime, household income and cohabitation status, caregiver's K6, and mother's age and employment.

Model 2: Model 1 with further adjustment for vegetable dishes, fish and meat dishes, milk and milk products, fruits, and confectionaries.
